# How Do Uterine Natural Killer and Innate Lymphoid Cells Contribute to Successful Pregnancy?

**DOI:** 10.3389/fimmu.2021.607669

**Published:** 2021-06-21

**Authors:** Oisín Huhn, Xiaohui Zhao, Laura Esposito, Ashley Moffett, Francesco Colucci, Andrew M. Sharkey

**Affiliations:** ^1^ Department of Obstetrics and Gynaecology, National Institute for Health Research Cambridge, Biomedical Research Centre, University of Cambridge School of Clinical Medicine, Cambridge, United Kingdom; ^2^ Centre for Trophoblast Research, Department of Physiology, Development and Neuroscience University of Cambridge, Cambridge, United Kingdom; ^3^ Department of Pathology, University of Cambridge, Cambridge, United Kingdom

**Keywords:** uterine natural killer cell, innate lymphoid cell, pregnancy, tissue resident natural killer cell, placenta, decidua, endometrium

## Abstract

Innate lymphoid cells (ILCs) are the most abundant immune cells in the uterine mucosa both before and during pregnancy. Circumstantial evidence suggests they play important roles in regulating placental development but exactly how they contribute to the successful outcome of pregnancy is still unclear. Uterine ILCs (uILCs) include subsets of tissue-resident natural killer (NK) cells and ILCs, and until recently the phenotype and functions of uILCs were poorly defined. Determining the specific roles of each subset is intrinsically challenging because of the rapidly changing nature of the tissue both during the menstrual cycle and pregnancy. Single-cell RNA sequencing (scRNAseq) and high dimensional flow and mass cytometry approaches have recently been used to analyse uILC populations in the uterus in both humans and mice. This detailed characterisation has significantly changed our understanding of the heterogeneity within the uILC compartment. It will also enable key clinical questions to be addressed including whether specific uILC subsets are altered in infertility, miscarriage and pregnancy disorders such as foetal growth restriction and pre-eclampsia. Here, we summarise recent advances in our understanding of the phenotypic and functional diversity of uILCs in non-pregnant endometrium and first trimester decidua, and review how these cells may contribute to successful placental development.

## Introduction

**Figure 1:**
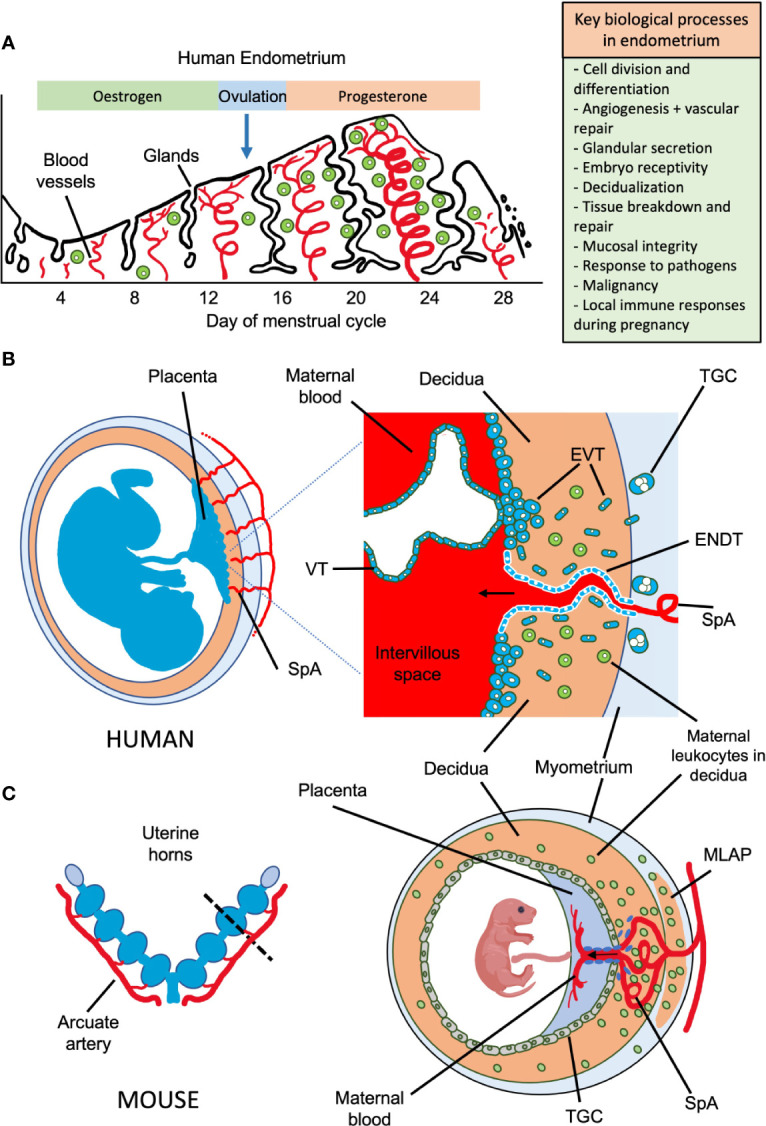
Possible roles of uterine NK cells and ILCs in human endometrium and placental development in humans and mice. **(A)** Important biological processes in endometrium during the human menstrual cycle are shown, which may be influenced by local immune cells. Following menstruation, the endometrium undergoes repair and proliferation under the influence of oestrogen from the ovary and blood supply is re-established involving both spiral arterioles and new capillaries. Following ovulation, progesterone action results in decidualization of the endometrial stroma and a rapid increase in the number of uNK cells, which comprise up to 70% of the leukocytes (shown in green) towards the end of the cycle. Dynamics of other ILC populations in human endometrium through the cycle are less well understood. Murine endometrium cycles by exposure to oestrogen then progesterone but the estrous cycle only lasts ~5 days and there is no menstruation. **(B)** The human maternal-fetal interface. Spiral arterioles (SpA) branch from the radial arteries and penetrate the decidua to supply the placenta. Right hand panel shows placental villi bathed in maternal blood which enters the intervillous space. Placental villi are covered by syncytiotrophoblast, beneath which is the villous cytotrophoblast layer (VT). Anchoring villi attach to decidua *via* columns of extravillous trophoblast (EVT) which differentiates from VT. Some EVT migrates down spiral arteries as endovascular trophoblast (ENDT) while other EVT cells migrate into the decidua as far as the myometrium where they transform into trophoblast giant cells (TGC). Interstitial EVT home to maternal spiral arteries and participate in their remodelling to provide a low pressure, high capacity blood supply to the placenta. During the first half of pregnancy, when spiral artery remodelling occurs, trophoblast invasion in humans is much deeper and more extensive than in mice. EVT invading into the decidua encounter maternal leukocytes (shown in green). In humans in the first trimester, these comprise: ~70% NK and ILCs, 20% macrophages and ~10% T-cells, which include CD4 and CD8 cells. Figure **(B)** adapted from Moffett and Colucci (1). **(C)** Schematic view of pregnant mouse uterus showing multiple implantation sites. Radial arteries branch from the arcuate artery to supply each developing fetus. The right hand panel shows a uterine cross section cut as indicated by the dotted line, with the arrangement of the placenta and decidua at gestation day (gd) ~12.5. The radial arteries penetrate through the myometrium, traversing a specialized structure that develops around mid-gestation, known as the mesometrial lymphoid aggregate of pregnancy (MLAP). The MLAP is not present in humans. Spiral arteries (SpA) branch from the radial artery into the decidua, rich in uterine NK cells but then merge at the interface between the placenta and the decidua to form a large blood canal that supplies the labyrinthine layer of the placenta where gaseous exchange takes place between maternal and fetal blood. This blood canal is lined with specialized fetal trophoblast cells (shown coloured purple). The boundary between the placenta and decidua is delineated by trophoblast giant cells (TGC), which show minimal invasion into the decidua - those few that do invade are largely perivascular. From gd12.5, glycogen rich trophoblast cells invade more extensively into the decidua (later than timepoint shown here), but spiral artery remodelling is largely complete by gd12.5. Panel **C** based on data from Adamson et al. (2).

The endometrial lining of the uterus is a highly unusual mucosal surface. It is a dynamic tissue that, in response to steroid hormones from the ovary, undergoes shedding, repair, extensive growth and remodelling up to 400 times between menarche and menopause (**Figure 1A**). If pregnancy occurs, the endometrium transforms into decidua to support implantation of the semi-allogeneic blastocyst and subsequent placental development (3). During the first trimester of pregnancy, decidual glandular secretions nourish the developing embryo. Extravillous trophoblast cells (EVT), derived from the blastocyst, invade into the decidua and remodel maternal spiral arteries to become high conductance vessels. These ensure a sufficient blood supply to the developing foetus from ~10 weeks gestation until term (4). This arterial transformation by trophoblast must be carefully regulated to avoid both extremes of insufficient or excessive invasion. Reduced invasion alters resource allocation between mother and foetus and is associated with common diseases of pregnancy including foetal growth restriction (FGR), pre-eclampsia, and miscarriage (5). Excessive invasion by trophoblast can also lead to pregnancy complications, for example when the placenta attaches over caesarean scars or in ectopic pregnancies. Thus, proper development of the maternal-foetal interface is a tightly controlled balancing act. The uterine immune system is involved in regulating this process.

Uterine natural killer cells (uNK) are the most abundant maternal immune cell in secretory phase endometrium and, if pregnancy ensues, constitute up to 70% of leukocytes in first trimester decidua. Numbers then decline substantially towards term (6). Non-NK ILCs are also present in the uterus, albeit in lower numbers, throughout the reproductive cycle. These include ILC1s, ILC3s and LTi-like cells, while the status of uILC2s is unclear. A unique feature of uILCs is that during pregnancy they can encounter trophoblast from a genetically different individual- the foetus. Therefore, uILC responses are regulated by interactions with maternal stromal cells, leukocytes in the decidua and with ligands expressed on EVT. Growing genetic and functional evidence suggests that interaction of uNK and other uILC subsets with EVT regulates the depth of foetal trophoblast invasion (1, 7). In addition, uILCs likely play important roles in homeostasis and tissue remodelling as well as in more canonical immune functions such as controlling pathogens and malignancies. Indeed, uNK can kill CMV-infected decidual stromal cells and are required for effective responses to *Chlamydia trachomatis* (8, 9). Thus, uILCs likely fulfil multiple functions depending on the context and their location (10). Moreover, these are likely to differ between species which have developed different mechanisms to sustain pregnancies. The maternal-foetal interface in human and mouse, and the potential roles uILCs play are summarised in **Figures 1B, C**. In both species, foetally-derived trophoblast cells invade the uterine mucosa during development of the placenta, although the extent of invasion is much greater in humans. This brings them into direct contact with decidual leukocytes, leading to the suggestion that uNK and uILCs regulate key processes during pregnancy.

The ability to perform a wide range of effector functions may be facilitated by heterogeneity within the uILC compartment. The most studied uILC are uNK, originally defined as CD3- CD56+ lymphocytes. There are several recent reviews of uterine ILCs (11–14). The diversity of uNK was apparent from the first phenotypic characterisations (15–17) and has become increasingly evident as high dimensional single cell technologies have been applied to the endometrium and decidua. Here, we focus on how these new approaches have expanded our understanding of the diversity of the uNK niche within ILCs and how these new uNK subsets may regulate normal placental development. We place recent findings in the wider context of ILC biology and discuss several outstanding questions that should guide future studies.

## NK and the ILC Family of Lymphocytes

**Figure 2:**
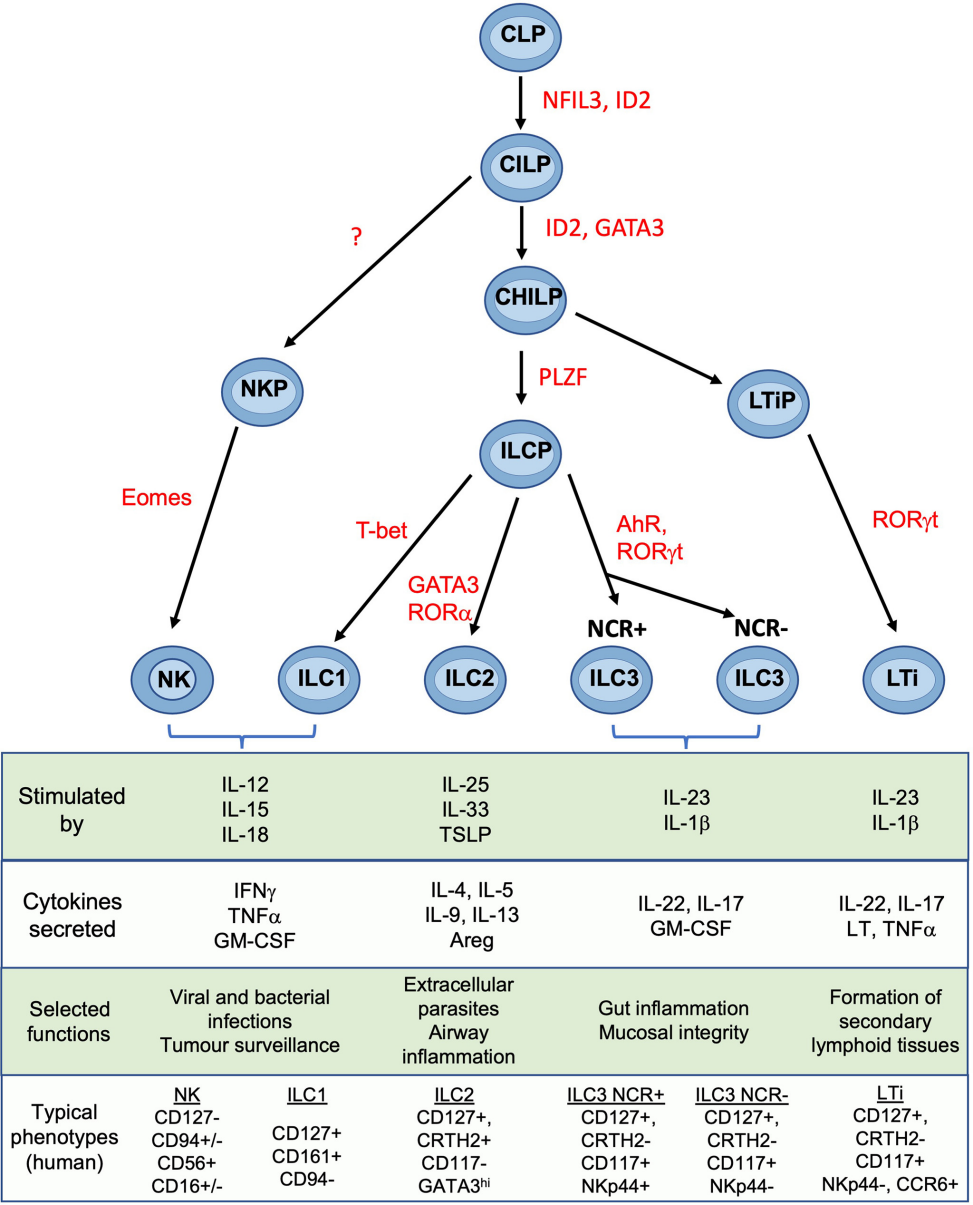
Development and functions of NK cells and ILCs Simplified overview of key stages of ILC development. Scheme shown is largely based on murine data. Table shows typical cytokines that stimulate the main ILC subsets, together with their principal effector molecules and known immune functions. Phenotypic markers commonly used to identify human subsets are shown and assumes prior gating on lin-CD45+ cells. CLP common lymphoid progenitor, CILP, common innate lymphoid progenitor; CHILP, common helper ILP; ILCP, innate lymphoid cell precursor; NKP, NK progenitor; LTiP, lymphoid tissue inducer precursor; LTi, lymphoid tissue inducer; NFIL3, Nuclear factor, interleukin-3 regulated; ID2, inhibitor of DNA binding 2; GATA3, GATA binding protein 3; PLZF, promyelocytic leukemia zinc finger; T-bet, T-box transcription factor 21; Eomes, eomesodermin; AhR, aryl hydrocarbon receptor; ROR, Retinoic acid–related orphan receptor; TSLP, thymic stroma lymphopoietin; Areg, amphiregulin; LT, lymphotoxin; IFNg, interferon-gamma; IL, interleukin; NCR, natural cytotoxicity receptor; NK, natural killer. Figure and phenotypic markers adapted from Vivier et al. (18) and Guia and Mancinelli (19).

NK cells are members of the ILC family which also contains ILC1, ILC2, ILC3 and lymphoid tissue inducer cells (LTi) (18). Lineage studies show that, in both mice and humans, NK cells and ILCs develop through a common lymphoid progenitor (CLP) but NK and ILCs then diverge (**Figure 2**). The latter proceed through a common helper-like ILC precursor (ChILP) dependent on the transcription factor PLZF (20). Subsequent development of the ILC1, ILC2 and ILC3 subtypes depends on differential expression of transcription factors T-bet, GATA-3 and RORγt respectively (21). In contrast, NK cell development from CLPs proceeds *via* an NK precursor and depends upon Eomes in combination with T-bet (20, 22–25). Several precursors have been identified that can develop into tissue ILC subsets. Identification of specific intermediates in human and murine ILC development is still underway and the mechanisms controlling ILC development may differ from the proposed scheme in certain tissues (26). Tissue ILCs also exhibit considerable plasticity within tissues, so the pathways leading to NK and other ILCs appear to vary in different organs (19). Tissue residency is a hallmark of ILCs, which like NK cells, lack antigen-specific receptors generated by somatic recombination. Although parabiosis experiments and constitutive cell tagging approaches in mice show that, under physiological conditions, ILCs and some NK subsets in non-lymphoid organs are largely tissue resident and maintained by self-renewal (27, 28), recent experiments suggest that ILC circulation is more extensive than previously thought (29). ILC3s can migrate from the intestinal mucosa to local lymph nodes and low numbers of ILCs circulate in the blood (30). Thus, the extent to which individual ILC and NK subsets can exchange with the circulation in different organs including the uterus is still unclear.

ILCs orchestrate context-specific immune responses and fulfil non-immunological roles including tissue remodelling and metabolic homeostasis (21, 31). Whilst the main ILC subtypes have common features across different organs, they acquire distinctive characteristics in response to local cytokines and the tissue microenvironment. Growing evidence for plasticity between some ILC subsets is also emerging, emphasising the need for detailed phenotypic and functional characterisation within each tissue (23, 32, 33). This variation has hampered efforts to define the identity and function of uterine ILCs.

## Uterine ILCs

Although all five main ILC subsets have been identified within the human uterus, the reports are somewhat conflicting and their exact phenotypic profiles and functional roles remain undefined (34–37). This is partly because studies of the functional properties of uterine ILCs and NK cells were based on phenotypic markers derived from other tissues. This can be problematic because of the phenotypic variability in different locations. Enzymatic disaggregation of tissue can also result in loss of key markers such as CD56 and NKp44; this is reduced by use of mechanical dissociation of tissue (35).

### Uterine NK Cells

NK cells were identified phenotypically as CD3−CD56+ granulated lymphocytes in blood (38). Large granular lymphocytes had been described early in the 20^th^ century in the uterine mucosa, but it was only with the advent of immunohistochemistry that these were shown to belong to the NK lineage (15–17). Studies over decades have relied on gating on CD3−CD56+ to capture NK cells, but this is now known to capture a mixture of both NK and non-NK ILCs in various tissues including the uterus (33, 37, 39, 40).

Endometrial NK Cells: NK cells in proliferative phase endometrium (eNK) comprise ~20% of total leukocytes. After ovulation, eNK then proliferate vigorously during the secretory phase which continues through early pregnancy (17, 41). This is driven by IL-15 secreted by stromal cells in response to progesterone (42). eNK are CD56^bright^, express markers of tissue residency (CD49a, CD69 and CD9), and many canonical NK cell markers (NKG2A, NKG2D and NKp46) but lack CD16 and CD57. The family of killer cell immunoglobulin-like receptors (KIR) are also important in regulating NK activation. KIR expression on eNK differs compared to matched pbNK as well as to dNK and is stable over multiple menstrual cycles (43, 44).

Compared with decidual NK cells (dNK), there are very few functional studies using eNK. They show cytotoxicity against K562 targets and low levels of spontaneous cytokine secretion, which is increased upon activation with IL-15 (41, 45, 46). Their rapid increase after ovulation has led to suggestions that eNK may be important in implantation. Accurate counting is difficult because of their uneven distribution and very rapid changes in number through the cycle, but numerous studies have failed to establish any consistent alterations in numbers of eNK and implantation failure or recurrent miscarriage (47). Nor is it clear that the wide variation in eNK numbers observed between these patients is functionally significant since it is also seen in women with normal fertility (48).

**Figure 3:**
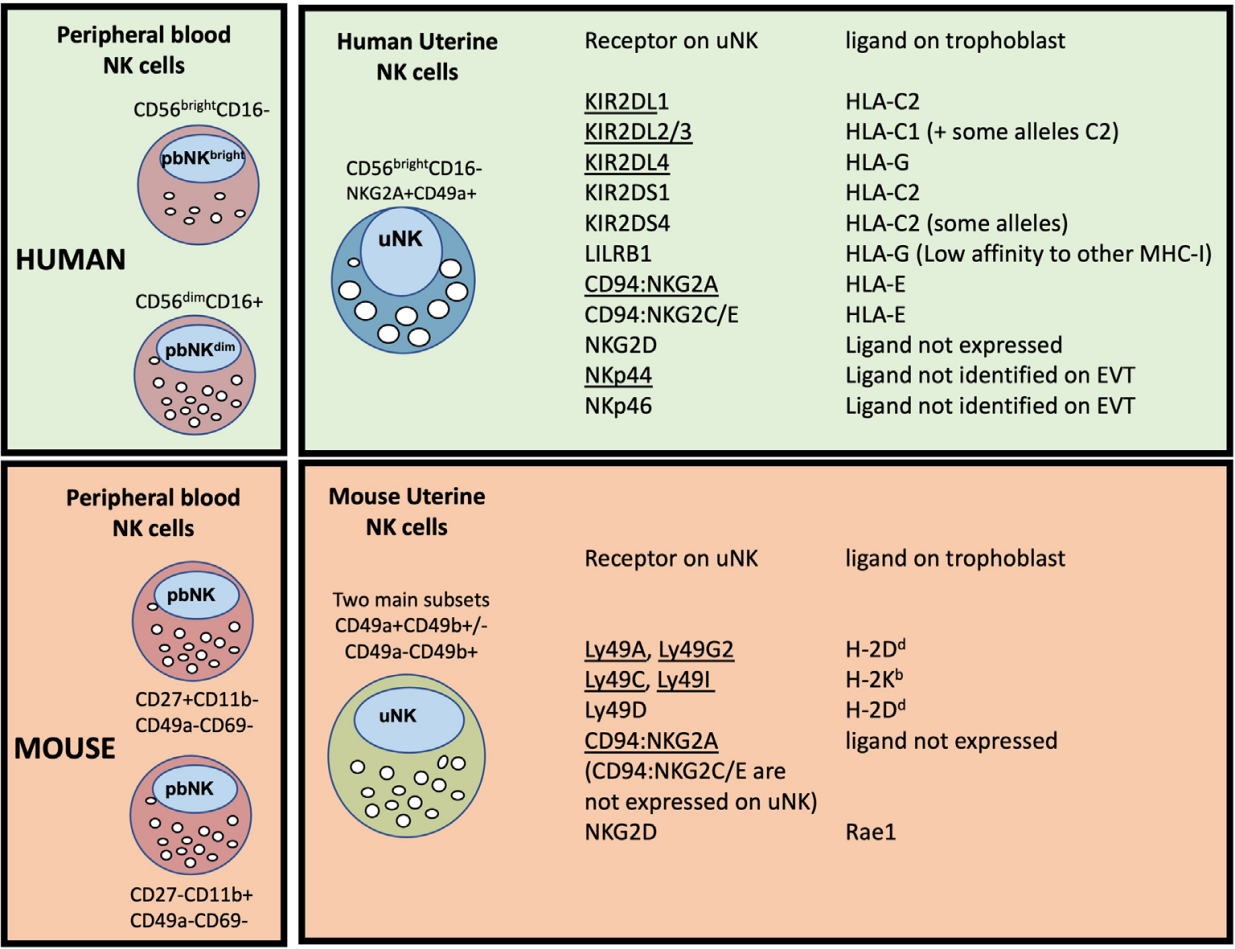
Major peripheral blood (pbNK) and uterine NK cell (uNK) subsets in human and mouse, with possible trophoblast MHC ligands for selected uNK receptors. The two main human pbNK subsets are Lin-CD56bright (~10% of total NK) and CD56dim (~90%); in mice the equivalent NK subsets are lin-CD27+CD11b+ and CD27-CD11b+ (56). In the human uterus, uNK cells are defined as lin-CD56+CD49a+ (or CD56+CD9+, since both CD49a and CD9 are markers of tissue residency in the uterus). Mouse uterine NK cells are typically gated as lin-NK1.1+NKp46+ and comprise two main subsets, distinguished on the basis of CD49a and CD49b expression (CD49b is often referred to as DX5). Recent studies have shown that uNK in both species exhibit further heterogeneity (described in more detail later). The right hand panel shows selected receptors expressed on uNK cells and whether the corresponding MHC ligands are expressed on human or murine trophoblast cells. Receptors that normally inhibit NK activity are underlined. Note that LILRB1 which is normally considered an inhibitory receptor, has been reported to function as an activating receptor in uNK cells by some authors (57, 58); conversely NKp44 may function as an inhibitory receptor in uNK (54), so is shown underlined. C2+HLA-C, indicates an HLA-C allele carrying a C2 epitope. Full details and original references describing expression of each ligand on trophoblast are available in Gaynor and Colucci (59).

First Trimester Decidual NK Cells: In comparison to eNK, first trimester dNK have been far more extensively studied. Phenotypically they share many similarities with eNK including expression of tissue residency markers, lack of CD16 and CD57, and increased proportion of cells that are KIR+ and NKG2A+ (43, 49–52). Comparison of eNK from the secretory phase with first trimester dNK, identified over 150 transcripts that differed >3 fold, highlighting significant changes that arise after the onset of pregnancy (53). As well as altered transcript levels, there are changes in RNA splicing, resulting in expression of inhibitory rather than activating isoforms of individual natural cytotoxicity receptors (NCR). Ligation of NKp30 or NKp44 therefore induces inhibitory responses in dNK but activation of pbNK (54). This may contribute to the low cytotoxicity displayed by dNK towards HLA class I null cell lines and to trophoblast (55). NK cells can mediate allo-recognition and dNK interact with EVT as the latter invade into the decidua. Because some of these receptors differ between mouse and human, the ligand/receptor interactions between dNK and foetal EVT are distinct in the two species, requiring caution in extrapolating results between the two (**Figure 3**).

Term Decidual NK Cells: As gestation proceeds, the proportion of dNK decreases and by term lin−CD56+ cells represent ~20% of CD45+ cells in decidua parietalis (60). They retain expression of CD9, lack CD16 and CD57, and display decreased expression of KIR, LILRB1 and 9kDa granulysin. There are also functional differences compared with their first trimester counterparts; term dNK degranulate more readily in response to PMA/ionomycin stimulation or K562 but show decreased cytotoxicity to HCMV-infected stromal cells. Thus, uNK are a dynamic population which vary in both function and phenotype depending on the stage of the reproductive cycle and location within the tissue.

### ILC1s

ILC1s, originally identified in the tonsils and gut mucosa, are Tbet**+** cells that produce IFN-γ in response to IL-12 stimulation (61). Two human ILC1 subsets have been described: “classical” ILC1s (CD56−CD94−CD127**+**CD117−NKp44−) and intra-epithelial ILC1s (ieILC1s) (CD56**+** CD103**+**) (62). The ILC1 cells identified in the decidua, resemble ieILC1s described in the gut, which express CD56, perforin and granzymes and are cytotoxic. ieILC1s express a unique integrin profile including CD103 and are found enriched in epithelial regions (62). ILC1s appear phenotypically heterogeneous and even display different transcription factor requirements for their development in different tissues so do not readily conform to the scheme outlined in **Figure 2** (63). In secretory phase endometrium no ‘classical CD127+ ILC1’ cells were detected (35) but both ILC1 and ieILC1 subsets are reported in decidua (36, 37). Indeed, NKp44+CD103**+** cells were identified as the major source of IFN-γ within the decidual Lin−CD56**+** compartment (64). Although CD56− ILC1s, ‘classical ILC1s’, are reported in term decidua, comprising ~3% of the Lin−CD127**+** compartment, their existence is controversial (64). The gating strategy used to identify ILC1s can capture a mixed population of other leukocytes and hematopoietic stem cells in some tissues (33). Current evidence therefore supports the presence of uterine ieILC1s but the presence of classical ILC1s needs confirming.

### ILC2s

Mixed reports also exist for the presence of uterine ILC2s, typically identified as Lin−CD127+CRTH2+ cells. A preliminary report showed ILC2s in small numbers in human non-pregnant endometrium and decidua and suggested they can promote foetal growth in mice (65). In human term decidua, ILC2s (Lin**−** CD56**−**CD127**+** GATA-3+) were the most frequent CD56**−** CD127**+** ILC, with increased ILC2 in decidua basalis of women with preterm labour (64). Other studies did not detect CRTH2 expression in endometrium or decidua (35, 36). These discrepancies may be explained by the gate used to identify ILCs. As found for ILC1s in the uterus, when gating on CD127+ cells, no ILC2s are detected in early decidua but CD127low/neg are fairly abundant (65). Whether cells corresponding to ILC2s exist in the uterus at all stages of the reproductive cycle is therefore controversial.

### ILC3s

ILC3s and Lymphoid Tissue-inducer-like cells (LTi-like) are defined by their expression of RORγt (66). LTi-like ILCs are phenotypically similar to LTi cells, which promote lymph node formation in the developing foetus. ILC3s can be further subdivided based on NKp44 expression into NCR**+** ILC3s and NCR− ILC3s; the latter are difficult to distinguish from LTi-like ILCs and have typically been treated as one population. ILC3s are described in both human and murine uteri (34, 35, 37). In human endometrium, the majority are RORγt+NKp44+ (~3% of leukocytes) corresponding to NCR+ILC3s (35, 37). Within the decidua, NCR**+**ILC3s produce IL-22 whilst NCR**−** ILC3s/LTis produce TNF upon activation (36). Both ILC3 and LTi subsets also induce the upregulation of adhesion molecules ICAM-1 and VCAM-1 on decidual stromal cells, suggesting they may be involved in recruitment of other leukocytes or can perform LTi-like functions (13, 36). Increased ILC3 proportions have also been detected in decidua parietalis of women with preterm labour (64).

These studies suggest that ILCs are represented across all stages of the human reproductive cycle and are dominated by uNK cells. However, the gating strategies needed to distinguish these subsets from one another in the uterine mucosa are not yet clear. For example, new findings that human dNK are CD56+CD94+ but heterogeneous for CD103 expression means it is problematic to identify ieILC1s, which in other tissues are typically gated as CD56+CD94+/−CD103+ (33, 37, 39, 40). For the same reason the presence of uterine ILC2s and classical ILC1s is not clearly established.

### Origin of Uterine ILCs

The dynamic changes in numbers of uNK and other uILCs in the endometrium and decidua has led to interest in where they originate. uNK cells have been the focus of most of these studies. Since NK cells originate from bone marrow derived precursors, work has centred on whether uNK cells derive from circulating CD34+ hematopoietic progenitor cells (HPCs) or NK lineage-committed precursors that migrate to the uterine mucosa. Others have examined whether mature blood NK cells can acquire uNK characteristics in the uterine microenvironment. Following bone marrow transfer, donor-derived leukocytes, including NK cells, are detected in endometrium and decidua in both humans and mice, suggesting that *in vivo* these can arise from transferred HPCs (67, 68). Human stage 3 NK-committed precursors can be detected in blood and can differentiate into more mature NK cells in the presence of IL-15, which is expressed in the endometrium and decidua (34). However CD34+ progenitors are also detectable in both human endometrium and decidua and can rapidly differentiate into NK cells *in vitro* (69). This suggests homing of NK precursors derived from bone marrow or differentiation of stem cells resident in the tissue may both contribute to uNK populations. The latter is supported by the finding that CD56+ NK cells develop in human endometrial tissue xenografted into immunodeficient mice, in response to steroid hormones (70). Mature human NK cells circulating in blood also acquire characteristics of uNK cells, after treatment with cytokines including TGF-β, suggesting that recruitment of pbNK can contribute to uNK populations (71). The idea that both *in situ* differentiation from tissue resident precursors and recruitment from cells circulating in blood contribute to uNK populations is supported by elegant parabiosis studies in mice. These show both mechanisms contribute to increases in uNK at different times: tissue resident cells proliferate during early pregnancy in response to decidualization, while a second wave of recruitment of circulating NK cells augments uNK cell numbers in the second half of gestation (72, 73). Recent data from HLA mismatched uterus transplant recipients in humans suggests that uNK can indeed be replenished from the circulation (74). This approach could be applied to establish whether other uILC subsets can also originate from the periphery and whether peripheral or tissue resident contributions vary during pregnancy, as seen in the mouse.

## High Resolution Analysis of Decidual NK and ILC Populations

It is now apparent that gating strategies used to define specific ILCs in one tissue may not readily translate to another because key markers can differ. Recently single cell RNA sequencing (scRNAseq) and high dimensional mass cytometry (CyTOF) have allowed ILCs and NK subsets to be reliably identified in different tissues despite this variation in phenotype and functional responses (33, 56). These techniques, as well as high parameter flow cytometry, have recently been applied to first trimester decidua to provide a more accurate characterization of the phenotype and functions of ILC subsets in the uterus (39, 40, 75–77).

Analysis by scRNAseq provided the first description of three distinct decidual NK populations, termed dNK1-3, as well as a proliferating NK subset (dNKp) and ILC3s (40, 77). dNK1 are characterised by expression of KIR, LILRB1, CD39 and increased granzymes. dNK2 express high levels of ITGB2 and anti-inflammatory ANXA1. Based on their expression signature, dNK3 resemble intra-epithelial ILC1s. No clusters corresponding to ILC2s or classical ILC1s were identified. This analysis also identified significant heterogeneity within other immune, stromal and epithelial cells and allowed the first systematic analysis of potential ligand/receptor interactions between uILCs and other decidual and placental cell types (40, 76, 78). scRNAseq analysis of luteal phase endometrium also identified several NK subsets suggesting that heterogeneity in uNK arises before pregnancy (79).

**Figure 4:**
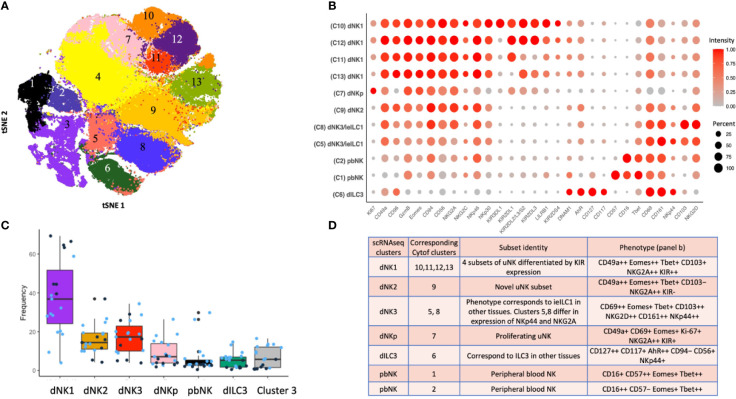
ILC subsets identified by mass cytometry of first trimester decidual cells. **(A)** tSNE landscape of CD45+ CD3- CD19- CD14- HLA-DR- (Lin-) cryopreserved decidual cells stained by mass cytometry. tSNE is coloured by clusters identified by DensVM clustering. 11 of these clusters can be assigned to NK or ILC subsets. Cluster 3 is heterogeneous and cluster 4 appears to contain cells damaged by cryopreservation so these were excluded from subsequent analysis. **(B)** Phenotypic characterisation of selected clusters. Size of the circle is representative of the proportion of the cluster positive for that marker. Circles are coloured by intensity of staining for that marker. Intensities have been scaled by marker (ie within each column). Red corresponds to higher expression and grey to lower expression. Percentage of cells in the cluster staining for each marker is indicated by size of the circles, scaled as shown on the right of the figure. **(C)** Box plots show the proportions of designated clusters within the total CD45+ Lin- decidual compartment. Blue dots = cryopreserved samples, Black dots = fresh samples. [**Figure 4A** adapted from Huhn et al. (39), where details of the antibody panel are described]. **(D)** Comparison of decidual NK and ILC clusters identified by single cell RNA sequencing (scRNAseq) (40) and by mass cytometry (Cytof) (39). Identity of each cluster is based on their profiles of RNA or protein marker expression respectively. Cytof clusters 10-13, correspond to scRNAseq cluster dNK1 and are distinguished by their KIR expression. Cytof clusters 5,8 are distinguished by NKp44/NKG2A expression and correspond to scRNAseq cluster dNK3, representing ieILC. Cytof clusters 3 and 4 identified in panel A appear heterogeneous and don’t match any scRNAseq clusters; identities are not established. ++ is > 75% positive staining of cells with antibody in Cytof; + is 25% to 75%; − is <25%.

A complementary analysis of lin−CD45+ decidual cells by CyTOF using a panel of 41 antibodies identified 13 separate clusters of which 11 can be phenotypically assigned to NK or ILC subsets (**Figures 4A, B**). This confirms the presence of multiple dNK subsets at the protein level (39). Clusters 10-13 (c10-13) correspond to dNK1 in the scRNAseq analysis but are further separated by differential KIR expression. Based on a similar receptor staining profile, including high expression of NKG2A and low expression of KIR and LILRB1, c9 corresponds to dNK2, and c5 and c8, which differ based on NKG2A and NKp44 expression, represent dNK3/ieILC1. Two small CD16**+** pbNK-like clusters (c1-2) are probably maternal blood contaminants as immunohistochemistry has revealed few CD56**+**CD16**+** NK cells within the decidua itself (17). dNK1 are the most abundant dILC followed by dNK2 and dNK3/ieILC1, although proportions vary between donors and can also be affected by cryopreservation (**Figure 4C**). These ILC subsets and their phenotypes are summarised in **Figure 4D**. Stimulation of ILCs by ‘missing self’ (K562) or PMA/ionomycin revealed that these dNK subsets significantly differ in their responsiveness, with dNK2 and dNK3/ieILC1 more responsive than dNK1. These are non-physiological stimulations and how each subset responds in more relevant assays such as co-cultures with trophoblast needs exploring.

In the light of these new findings a re-evaluation of previous studies using pre-determined gating strategies to identify dNK subsets is possible. Three populations were found using NKp44 and CD103: NKp44+CD103+, NKp44−CD103+ and NKp44−CD103− (37). Although NKp44+CD103+ are enriched for dNK3/ieILC1, the remaining subsets do not directly correlate with those defined by CyTOF. pbNK cells can display memory-like properties with expansion of specific NK subsets in CMV infection (80). Expansions of uNK subsets have been described in both mice (81) and humans (44, 57, 82). A population of NKG2C^high^ LILRB1**+**, termed pregnancy-trained decidual NK (PTdNK) is increased in secondary and subsequent pregnancies. Phenotypically, these most closely resemble dNK1 and probably represent an expanded dNK1 subpopulation. PTdNK have epigenetic and transcriptomic profiles that favour IFN-γ and VEGF-A production although the functional studies were performed with dNK activated after IL-15 priming (57). IFN-γ and VEGF-A are not normally produced by freshly isolated dNK1 unless they are strongly activated after isolation, so their role *in vivo* is unclear (83, 84). First pregnancies show a higher risk of low birth weight and pre-eclampsia, which may be due to better extra-villous trophoblast invasion and arterial transformation in subsequent pregnancies (85–87). Further work is required to define the PTdNK subset more accurately to ascertain if they contribute to this effect.

### How Do Decidual ILC Populations Compare With Other Tissues?

**Figure 5:**
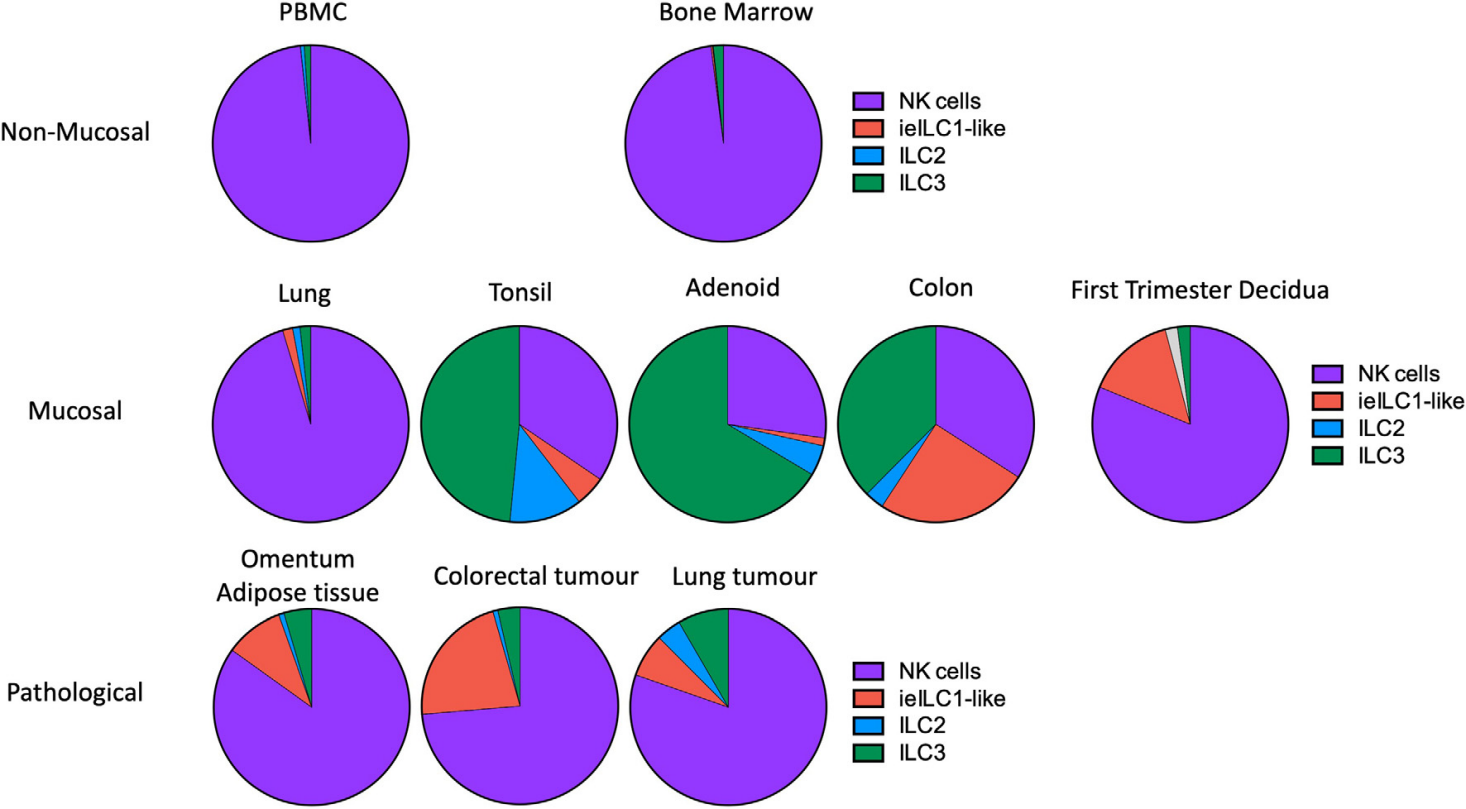
Frequencies of NK and ILC populations in decidua compared with other human tissues. Pie charts showing the frequencies of NK and ILC subsets in decidua (N = 7) compared with normal and pathological human tissues determined by mass cytometry. All charts, with the exception of first trimester decidua come from Simoni et al. (n = 4-9 separate donors for each tissue type) (33). For first trimester decidua, Live CD45+CD3-CD19-CD14-HLA-DR- cells were gated on as the parent population [data from Huhn et al. (39)]. Specified subsets were then identified on a tSNE landscape based on phenotypic profiles and their proportion within this population calculated. Unassigned cells (grey in decidua) could not be confidently labelled as a particular subset based on the markers included in the CyTOF panel. For all other tissues, samples were first depleted of T and B cells and then gated on Live CD45+ FcϵR1α– CD14– CD19– CD123–CD34-CD5- cells. Gating strategies to identify each subset were then as follows: NK cells (purple) = CD94+/-CD127+/-CD56+CD103-; ieILC1-like (red) = CD94+/-CD127+/-CD56+CD103+; ILC2 (blue) = CD94-CD127+CRTH2+; ILC3 =CD94-CD127+CRTH2-. [Figure adapted from Simoni et al. and Huhn et al. (33, 39)].

ILC subset composition varies between tissues and pathological states but in first trimester decidua it differs from other normal mucosal tissues (**Figures 4C**, **5**) (33, 39). Some caution is needed with this comparison as the gating strategies used in the two studies are not directly comparable. The lung contains relatively fewer ieILC1 cells whilst colon, adenoid and tonsil all possess larger proportions of ILC3s than decidua. Intriguingly, in this analysis the decidual ILC composition more closely resembles that seen in colorectal tumours with NK and ieILC1-like cells predominant and few ILC3s (**Figure 5**). This relative lack of ILC3s in decidua is consistent with a recent analysis of normal mucosal tissues, which also demonstrated significant variability in ILC composition at different sites within the same tissue (63). dNK1 are unlike tissue resident NK (trNK) present elsewhere; their expression of high levels of both KIR and NKG2A is unique (33, 88). For example, liver resident NK (lrNK) subsets are defined by CD49a and CXCR6. However, CD49a+ lrNK are KIR+ NKG2A− and CXCR6+ lrNK are KIR− NKG2A+ (89–91). Similarly, dNK2 may resemble CXCR6**+** lrNK with respect to KIR and NKG2A expression but differ in terms of CD49a expression. In contrast, dNK3/ieILC1 and dILC3 more closely resemble their counterparts in other tissues. The presence of multiple uNK subsets is suggestive of subset-specific functions.

### Microheterogeneity Within uNK Subsets

**Figure 6:**
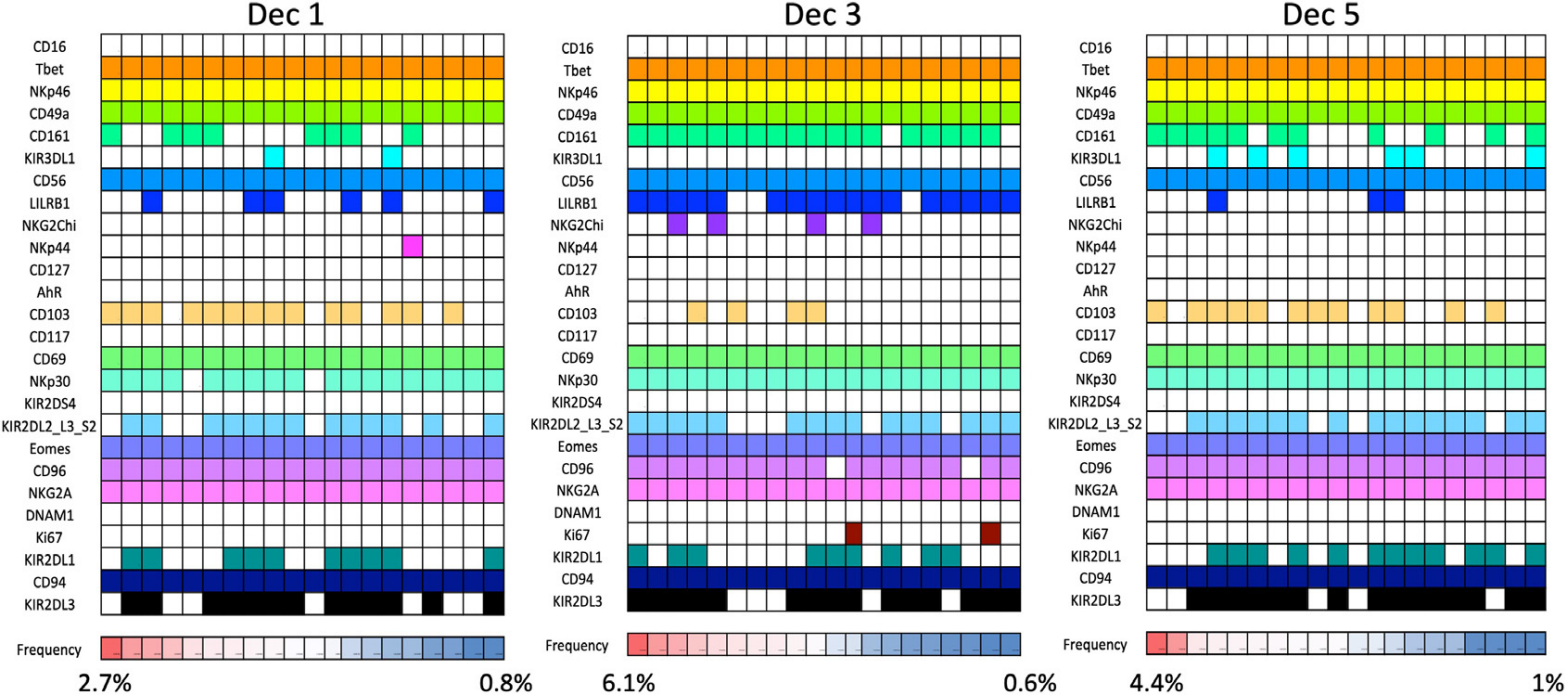
dNK receptor repertoire diversity Frequencies of the most common NK cell phenotypes were determined by mass spectrometry in freshly isolated CD45+Lin−CD56+ decidual NK cells for three selected individuals based on combinations of 26 individual phenotypic markers expressed on each cell. The twenty most frequent phenotypes detected are shown for each donor Dec1,3,5. Each column represents a phenotype shared by a number of cells; boxes are coloured if the marker is expressed in that phenotype. Frequencies of each phenotype as a percentage of total CD45+Lin−CD56+ are displayed as a heat map at the bottom of the figure. Based on analysis of data from Huhn et al. (39).

Our mass cytometry analysis has revealed further phenotypically discrete populations within these novel uNK subsets. Many NK receptors are expressed stochastically, further diversified by environmental factors including NK cell education, epigenetics and viral infections as well as genetic differences between individuals (92, 93). Using Boolean analysis of receptor combinations, pbNK exhibit a large level of receptor diversity within the main CD56^bright^ and CD56^dim^ fractions (94). A similar analysis of CD45+ Lin− CD56+ cells using our CyTOF data reveals comparable diversity within the decidual NK subsets (**Figure 6**). 25 additional markers were included so 2^25^ phenotypic combinations are theoretically possible. For pbNK, up to 6173 phenotypes were identified for a single donor using 28 markers, with the most frequent phenotype present representing ~0.6-4.6% of total pbNK from six donors. Subsets comprising the most frequent 20 phenotypes accounted for between 6.1-25.9% in each donor’s pbNK (**Table 1**). For dNK, up to 4745 phenotypes were detected for a single donor. In contrast to pbNK, the top 20 phenotypes represented a larger proportion of the total NK compartment, ranging from 25.2-44.3% between donors. Cells expressing KIR (mainly corresponding to dNK1) occur more frequently in the most common dNK phenotypes in comparison to pbNK. While overall phenotypic diversity appears similar in pbNK and dNK, the decidual NK cell repertoire is dominated by a more limited number of highly represented phenotypes, possibly selectively favoured during NK proliferation and maturation. Some may represent developmental intermediates while others include effectors that drive important physiological processes during pregnancy. The immediate challenge is to determine how the phenotypic diversity of uNK and ILC populations within and between women influences pregnancy outcomes.

**Table 1:** Phenotypic diversity of CD45+Lin−CD56+ cells in human blood and decidua based on receptor profiles of individual cells analysed by mass cytometry.

Donor	Tissue	Input number of cells	Number of phenotypes	Frequency of most frequent NK phenotype^1^(%)	Total frequency captured by 20 most frequent phenotypes^1^(%)
Pb1	Blood	25683	6173	1.9	18.6
Pb2	Blood	9736	4509	1.9	13
Pb3	Blood	7892	3563	2.2	14.5
Pb4	Blood	10787	3336	4.6	25.9
Pb5	Blood	4164	2882	0.6	6.1
Pb6	Blood	14483	4673	2.2	19.6
Dec 1	Decidua	24031	3792	2.7	25.2
Dec 2	Decidua	24740	4428	3.8	32.5
Dec 3	Decidua	32197	4745	6.1	36.8
Dec 4	Decidua	24670	3206	5.6	36.2
Dec 5	Decidua	32507	3658	16	44.3
Dec 6	Decidua	32935	3261	4.4	31.4

^1^Frequencies expressed as % of total CD45+Lin−CD56+ cells in blood or decidua. Blood and decidual samples not from matched donors. Data derived from Huhn et al. (39).

## What Can We Learn About the Function of Uterine ILCs From Mouse Models?

Establishing the function of human NK cells and ILCs is challenging because of the rapid changes in cell types and ongoing uncertainty about which ILCs are present at different stages of pregnancy. Implantation and early placental development in the mouse do share some features with humans, including decidualisation of stromal cells of the uterine mucosa, and invasion of foetal trophoblast cells into the decidua (**Figure 1** and **Table 2**). Although trophoblast invasion and spiral artery remodelling is necessary in both species to increase the blood supply to the developing placenta, this invasion is far more extensive in humans. Human trophoblast invades both down the blood vessels (endovascular) and through the decidua (interstitial) extending as far as the inner myometrium (**Figure 1B**). Trophoblast cells mediate the classical fibrinoid necrosis of the arterial media that transforms these vessels (95, 96). In mice, trophoblast invasion is minimal and limited to the junctional zone of the placenta during the first 12 days of gestation. Instead, spiral artery modification is associated with NK cells which move into the arterial wall (59, 97, 98). Nonetheless, invading trophoblast cells do encounter maternal NK and ILCs in the murine decidua later in pregnancy after spiral artery remodelling is completed.

**Table 2:** Key Features of human and murine pregnancy models.

Description	Human	Mouse
Duration	40 weeks	~19 days
Implantation sites	Typically one	Multiple implantation sites in each uterine horn
Decidualisation of endometrial stromal cells	Begins in non-pregnant endometrium. Progesterone dependent	Triggered by embryo at site of attachment. Also progesterone dependent
Uterine NK cell numbers	Proliferate in endometrium before pregnancy Increase rapidly in first trimester then decline	Few in endometrium
		Rapid increase in trNK after implantation starting ~ gestation day (gd) 4.5
		Peak at mid-pregnancy ~gd 10 then decline
Trophoblast invasion	Extensive: through decidua and even into myometrium by:	Limited: to junctional zone until gd12.
	Interstitial trophoblast, Endovascular trophoblast, Placental bed giant cells	Limited invasion of decidua thereafter by:
		Trophoblast giant cells and glycogen cells
Vascular remodelling	Associated with endovascular trophoblast in lumen.	Associated with NK cell intravasation into smooth muscle of vessel
	Interstitial trophoblast in muscle wall.	Mediated in part by IFNγ from NK cells
	Some evidence for NK involvement before trophoblast invasion and away from placental bed	Endovascular Trophoblast limited to central canal in placenta
Localisation of NK cells	Isolated cells and in aggregates In pregnancy, is limited to decidua, Not in myometrium	NK cells in decidua and in the MLAp, embedded in the myometrium

(MLAp is the mesometrial lymphoid aggregate of pregnancy, not seen in humans).

There are also important differences in timing as well as localisation of ILC populations. Decidualization is always characterized by extensive accumulation of NK cells in both species, but in humans this process begins in the endometrium, so large numbers of proliferating NK cells are present even before pregnancy begins. In the mouse, NK numbers do not increase until decidualization is triggered by implantation of the embryo. In both species NK cells increase rapidly during the first trimester but decline by term (6, 11). In mice, at mid-gestation, ILCs are located in the mesometrial lymphoid aggregate of pregnancy (MLAp) which develops in the myometrium (11). No such structure exists in humans and the function of the MLAp is unknown. It may participate in the regulation of blood flow since the uterine artery that supplies each implantation site coils through it (99). In addition, NK-derived IFNγ is a key cytokine in murine pregnancies but it is not a major cytokine produced by the main dNK1 subset in humans. Despite these apparent differences in anatomy and cellular dynamics, human and murine pregnancy share key features. These include trophoblast invasion into the decidua and the involvement of both foetal trophoblast and maternal uNK cells in spiral artery remodelling to ensure sufficient blood reaches the haemochorial placenta. This suggests mice can provide a useful model to study the role of NK cells and ILCs in placental development.

**Figure 7:**
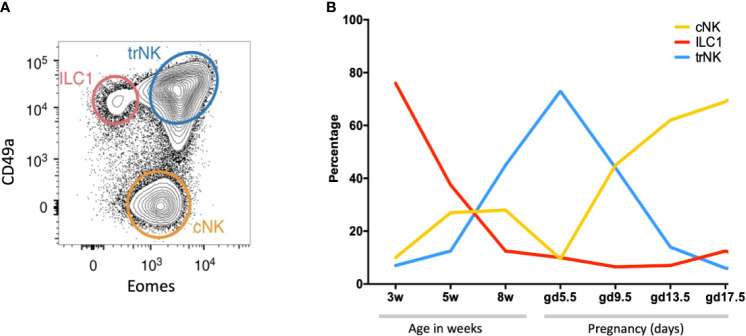
Dynamic changes of NK and ILC1s in the mouse uterus before and after pregnancy **(A)** Gating strategy to identify uterine NK and ILC1 subsets. Cells isolated from whole uterus were analysed together. Representative 2D FACS plot is from gestational day 9.5 (gd 9.5) and gated on Live CD45+CD3−CD19−CD11blow/- NK1.1+NKp46+ cells. ILC1s (red line) are CD49a+ Eomes−, tissue resident NK (trNK, blue) are CD49a+Eomes+, conventional NK (cNK, gold) are CD49a− Eomes+. **(B)** Proportions of indicated uterine subsets at different stages of the reproductive cycle as a percentage of the total Live CD45+CD3−CD19−CD11blow/- NK1.1+NKp46+ cells. w = weeks of age, gd = gestational day [Figure adapted from Filipovic et al. (81)].

NK cells and ILC1s are the most abundant leukocytes in the pregnant mouse uterus (~30% of total leukocytes at mid-gestation), significantly lower than in human first trimester decidua (35). There are three subsets: tissue resident NK (trNK), CD49a+Eomes+, conventional NK (cNK), CD49a−Eomes+, and ILC1s, CD49a+ Eomes− (**Figure 7A**). These all change rapidly in number and location throughout the reproductive cycle (**Figure 7B**). Prior to puberty, mouse endometrium contains predominantly ILC1s. These decline after puberty while cNK and trNK increase (81). ILC2s and ILC3s are also present in low numbers prior to pregnancy, the latter comprising both LTi-like and NCR+ ILC3s (35, 100). ILCs are distributed throughout the tissue in a subset-specific manner. At gestational day 11.5 of pregnancy (gd 11.5), the decidua basalis and MLAp are composed of mainly cNK or trNK respectively. ILC2 and ILC3s are absent from the decidua (35, 36). As outlined when discussing the origins of uNK and ILCs, different rates of subset proliferation, influx from blood or conversion between ILC subsets within the uterus may all contribute to these rapid changes in cellular composition.

Relating human decidual ILC subsets to their murine counterparts is difficult. dNK1 may be analogous to trNK that express high levels of Ly49, Eomes and Ki-67 (28, 81). Murine cNK resemble circulating NK in humans but cNK are present in large numbers in murine decidua whilst CD16+ pbNK are rarely seen in human decidual tissue (17, 35, 73). Murine uILC1 expand in secondary pregnancies and express CXCR6, a hallmark of antigen-specific NK memory cells (101). Thus, they may be the functional counterparts to human PTdNK ‘memory’ cells but differ in their low Ly49 expression (57, 81). How human dNK2 and dNK3/ieILC1 relate to murine decidual ILCs is also unclear. Murine ieILC1 in the gut are Nfil3-dependent NKp46+ NK1.1+ CD160+ (62). However, *Nfil3^-/-^* mice contain both uterine trNK and ILC1 but lack cNK cells (100). The difficulties in directly correlating murine and human dILC subsets may reflect the anatomical differences between human and mouse placentation as well as the menstrual versus oestrus cycles in which ILCs and uNK develop preceding pregnancy. Nevertheless, further characterisation may reveal functional homologies between phenotypically distinct uterine ILC subsets between the species.

## Functions of Uterine ILC Populations During Pregnancy

Evidence highlighting the importance of uNK cells in supporting successful pregnancies has come from both humans and mice. Loss of functional NK cells and/or ILCs in a variety of mouse models is associated with decidual abnormalities and reduced vascular remodelling (102) and foetal growth restriction (98, 100, 103, 104). For example, pregnancies from an NK- and T cell deficient mouse model, *tg*ϵ*26*, exhibit smaller litters and lower birth weights. Normal birth phenotypes could be restored by bone marrow grafts from SCID mice that lack B and T cells but possess NK cells (105). These changes are in part due to loss of IFNγ secretion by NK cells, since they are rescued by systemic IFNγ administration and similar effects are seen in mice lacking IFNγ or its receptor (102, 103). Even deletion of single receptors on maternal NK cells can result in abnormal foetal development. Mice lacking Nkg2a, Ncr1 or AhR show defective NK cell maturation, abnormal spiral artery remodelling and lower foetal weights (106, 107). Depletion of NK cells in pregnant rats also causes abnormal arterial remodelling and hypoxia, accompanied by differentiation of more invasive trophoblast resulting in abnormal placental development (108). The roles of other ILCs in pregnancy are much less well understood. Mice lacking the transcription factor Nfil3/E4bp4 show greatly reduced numbers of cNK and ILC2s in the uterus but retain trNK, ILC1 and ILC3s. This is associated with placental abnormalities and reduced foetal growth indicating that the altered ILC repertoire affects the outcome of pregnancy (100). The foetal growth restriction in *Nfil3*-/- mice is reversed by infusion of pleiotrophin, supporting the idea that NK or ILC subsets produce factors in addition to IFNγ that promote placental development, but the detailed mechanisms responsible are unclear (104).

### Regulation of Trophoblast Migration

A unique feature of pregnancy is that dILCs interact with EVT from the foetus as well as with maternal cells in the decidua. Following implantation, in both humans and mice, foetally-derived trophoblast migrate into the decidua where they encounter maternal immune cells. These foetal cells express an unusual repertoire of MHC class I antigens: human EVT is negative for HLA-A and -B but expresses polymorphic HLA-C and oligomorphic HLA-E and HLA-G molecules (109). Trophoblast are always MHC class II negative. Murine trophoblast from C57BL/6 mice express the MHC class I antigen H-2K^b^ but very low levels of H-2D^b^ and Qa-1b. Many of the receptors on uNK that recognise these polymorphic MHC molecules are also highly variable and include members of the killer cell immunoglobulin-like receptors (KIRs) in humans or the Ly49 family in mice (**Figure 3**). The dNK1 subset in particular expresses relevant receptors for trophoblast HLA molecules, including NKG2A, LILRB1 and members of the KIR family (**Figures 3**, **4B**). Their activation status can therefore be influenced by both foetal and maternal ligands. Maternal KIR recognition of HLA-C molecules on EVT is of particular interest because both KIR and HLA-C genes are highly polymorphic so each pregnancy is characterized by different combinations of these genes, resulting in variable activation or inhibition of dNK. Genetic studies of large pregnancy cohorts suggest that women with a KIR AA genotype are at increased risk of pregnancy disorders when the foetus has an HLA-C allele carrying a C2 epitope (C2+HLA-C). This combination results in strong inhibitory signals to uNK *via* maternal inhibitory KIR2DL1 binding to foetal C2+HLA-C on EVT and is associated with increased risk of pre-eclampsia, foetal growth restriction and miscarriage, although not all studies concur (110–113). In contrast, combinations of maternal KIR and foetal HLA-C which favour activation through KIR2DS1 or KIR2DS5 are associated with protection from pre-eclampsia and increased birth weight (110, 112, 114–116).

These are all disorders of pregnancy in which EVT migration and vascular remodelling is reduced (5). More recently the presence of the strongly inhibitory allele KIR2DL1*003 in mothers, combined with foetal C2+HLA-C ligand has been shown to further increase risk in a dose dependent manner (117). Thus, binding of a specific inhibitory receptor expressed on uNK with the corresponding ligand on EVT can affect pregnancy. These effects are likely to be mediated by dNK1 since they are the main KIR-expressing cells (**Figure 4B**). This idea is supported in a mouse model in which the addition of a single additional MHC molecule (H-2D^d^) to the foetal genome results in abnormal uterine artery remodelling and reduced foetal growth. This suggests that maternal recognition of a single paternally-derived H-2D^d^ molecule influences pregnancy outcome (98). Whether the mechanism involves direct recognition of paternal H2-D^d^ expressed on trophoblast by inhibitory Ly49 receptors on maternal dNK cells, as seems to occur for human dNK1 and C2+HLA-C on EVT, is not yet certain. In humans, the role of other dILC subsets is less clear. dNK2 and dNK3/ieILC1 express receptors for HLA-E and HLA-G and other ligands on trophoblast, but these are not significantly polymorphic, so will not vary between pregnancies (39, 40). dNK2 and dNK3 respond vigorously to activation, but how they respond to EVT *in vivo* and how the resulting cytokine responses contribute to pregnancy success, is not known. Patients with SCID due to mutations in *IL2RG* or *JAK3*, have very low levels of ILC and NK cells in blood, gut and skin after hematopoietic stem cell transplantation (HSCT). Despite this, two women had clinically normal pregnancies after HSCT and healthy babies of average weight (30). While this study did not investigate uterine ILCs in these women, the results suggest NK cells and other ILCs are not essential for reproduction under normal conditions, but can influence pregnancy outcome by fine-tuning placental development and hence foetal growth. This is likely achieved by fulfilling multiple functions including regulating trophoblast invasion, and remodelling of the vasculature and decidual tissue.

**Figure 8:**
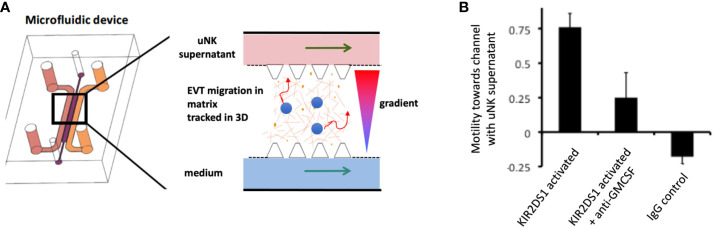
Ligation of activating KIR on uNK affects human trophoblast migration *in vitro.* **(A)** Cartoon showing a cross section of a microfluidics device to study effects of uNK supernatants on migration of extravillous trophoblast (EVT) . Primary human EVT isolated from first trimester placentas are stained with tracker dye (blue) and embedded in Matrigel in the central channel. Cells are tracked in real time as they move between two side channels. These side channels have medium constantly flowing through them and can be supplemented with other factors or supernatants conditioned by uNK cells to create a gradient. **(B)** Plot showing motility of EVT towards or away from a microchannel containing uNK-conditioned medium. Freshly isolated uNK were purified from decidua using microbeads and KIR2DS1 was activated by EB6 antibody cross-linking for 12 hours. Stimulation conditions are shown on x-axis. On average, EVT display increased motility towards a channel containing supernatant from uNK stimulated by KIR2DS1 cross-linking. This is reduced by the addition of neutralising anti-GM-CSF in the supernatant. Control is ligation with isotype matched irrelevant IgG. Motility is calculated by subtracting the number of cells migrating upstream towards the uNK supernatant channel by the number of cells migrating away and dividing this by the total number of cells, [Figure adapted from Abbas et al. (120)].

What are the critical responses triggered by KIR in dNK that might affect placental development and pregnancy outcome? dNK produce a wide array of chemokines and cytokines upon stimulation with PMA plus ionomycin, or by ligation of activating receptors (37, 52, 118, 119). Ligation of activating KIR to simulate dNK/EVT interactions in the decidua stimulates secretion of GM-CSF and CXCL10, which have been shown to increase EVT migration (52, 115, 119). In a 3D microfluidics model, primary trophoblast migrate towards a microchannel containing uNK-conditioned medium following activation of KIR2DS1 (**Figure 8**) (120). uNK have also been shown to secrete TGF-β which reduces EVT migration and this effect changes with gestational age, so uNK responses can potentially enhance or decrease EVT migration (121). Whilst no migration model can replicate the complex tissue environment of the decidua, these genetic and functional studies suggest ligation of activating KIR on dNK can modulate EVT behaviour and thus affect blood flow to the placenta.

**Figure 9:**
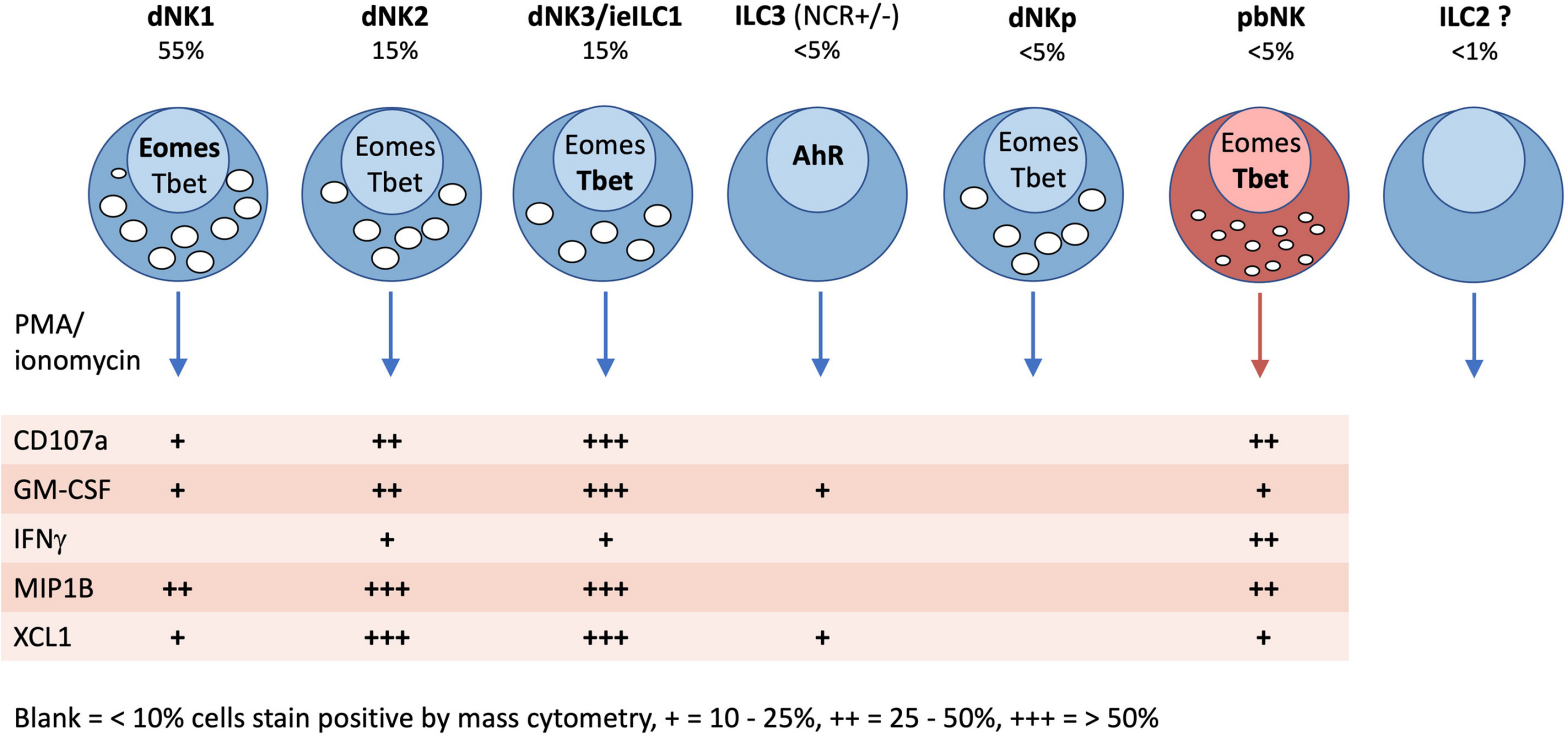
Characteristics of innate lymphoid cells identified in the human uterus by scRNAseq and mass cytometry. Typical frequencies of each ILC type are given as a percentage of total decidual CD45+HLADR −CD14−CD19−CD3− (Lin−) gate in freshly isolated cells (not cryopreserved). Frequencies in endometrium will be different. Transcription factor (TF) expression and cytokine responses following PMA/ionomycin stimulation are based on mass cytometry data of Huhn et al. (39). TFs and cytokines listed in bold, indicate stronger staining. For functional responses, blank = 0-10%, + = 10-25%, ++ = 25-50%, +++ = 50+% staining by mass cytometry.

### Vascular Remodelling in Decidua and Non-Pregnant Endometrium

The role of uNK-derived IFNγ in vascular remodelling in murine decidua is well established (102). Impaired vascular remodelling in the decidua of mice lacking NK cells or specific NK receptors, early in gestation shows that uNK cells may directly participate in this process prior to the arrival of trophoblast (122). This may be a direct effect on the arteries as they infiltrate the media, unlike in humans where uNK are seen around the arteries but not in their wall. Murine uNK also secrete VEGF-C and mice lacking the corresponding receptor VEGFR3 on endothelial cells show reduced vessel remodelling and foetal growth restriction. This shows soluble factors secreted by uNK in mice can target endothelium lining the arteries, as well as the surrounding smooth muscle (123). There is indirect evidence that uNK may also influence spiral arteries around which they are seen to cluster in human endometrium and decidua (95, 124). dNK produce MMP-9 which breaks down extracellular matrix (ECM) of the vascular smooth muscle wall and angiogenic factors such as VEGF-C and angiopoietins-1 and -2 (119, 125–127). Although these could disrupt the vascular smooth muscle wall, as their effect is partially abrogated by Ang-2 inhibition using *in vitro* models, little is known about the mechanisms by which human uNK or EVT actually influence vascular remodelling *in vivo* (121, 128, 129). Evidence that NK cells can play a role in regulating the vascular remodelling of the human endometrium prior to pregnancy is more compelling. Clusters of eNK around spiral arteries express angiogenic factors including VEGF-C and PLGF which can regulate endothelial cell function (125). Administration of a progesterone modulator (Asoprisnil) to women results in absence of eNK, altered arterial morphology and no menstrual bleeding (42). Asoprisnil blocks IL-15 secretion by stromal cells in response to progesterone. Whether the outcomes are due to the reduced uNK or other uILCs or a consequence of blocking other actions of progesterone is unclear. The role of uNK in modifying the arterial media needs exploring further as the signals triggering menstruation and decidual breakdown in miscarriage are still essentially unknown (129). One suggestion is that eNK use the activating receptor NKG2D to kill senescent stromal cells emerging during the secretory phase (130). NK cells kill senescent cells in tumours and in mouse models of liver fibrosis, but whether this is important for homeostasis in cycling human endometrium *in vivo* needs verification (131, 132).

### Regulation of Local Immune Responses in the Decidua

It is clear that dNK can recognise both maternal and paternal HLA-C expressed by invading EVT, but allogeneic trophoblast are also potential targets for maternal T cells. dNKs have been shown to secrete cytokines and chemokines which have immunoregulatory functions, leading to suggestions they contribute to foetal-specific T cell tolerance in the decidua *via* a plethora of mechanisms (133, 134). For example, dNK activated *in vitro* to secrete IFNγ, drives IDO production by decidual CD14**+** cells and induces expansion of regulatory T cells (Treg) (135). A caveat is that many functional *in vitro* studies use dNK stimulated by unphysiological levels of IL-15 or other factors and their relevance is questionable. Freshly isolated dNK typically produce low levels of IFNγ and other cytokines, unless strongly activated after isolation (83, 126, 136), and decidual stromal cells have also been reported to secrete IFNγ (137). New approaches such as scRNAseq and spatial transcriptomics are required to investigate how uNK influence responses of other immune cells within the decidua *in vivo*.

Recently, the ectonucleotidase, CD39, was found to be expressed by dNK1; this could combine with CD73 on EVT to convert ATP to adenosine, which is associated with local immune suppression (40, 74). Trophoblast-specific T cells are likely to be HLA-C restricted, since this is the only polymorphic HLA-molecule on trophoblast. In term decidua, Tregs are induced following expansion of T cells in the decidua of HLA-C mismatched pregnancies (138). Many potential mechanisms favouring T cell tolerance in the decidua have been demonstrated *in vitro*, but whether effector T cells ever cause foetal loss in humans, and if dysfunction of dNK or other dILCs contribute to this, is not yet established (1, 133, 139).

Despite the immunomodulatory environment in the decidua, maternal immune cells including ILCs must be able to respond to pathogens. For example, uILC3s produce IL-22 which maintains mucosal integrity, and has been shown to maintain pregnancies in a mouse model of infection-induced pregnancy loss (140). dNK can also counteract infections directly; they inhibit infection of human decidual macrophages by HIV *in vitro* and kill decidual stromal cells infected with HCMV (141). dNK expressing an activating KIR were more cytotoxic against infected decidual stromal cells than dNK lacking that activating receptor. This effect was enhanced when the stromal cells also expressed the cognate ligand for the activating KIR (8, 83). dNK have also been shown to suppress infection by *Listeria monocytogenes* of primary trophoblast cells and decidual macrophages without killing the infected cells (142). Exactly which dNK or dILC subsets are responsible for these antipathogen effects is not established. Thus, uILCs contribute through multiple redundant mechanisms to enhance the resilience of pregnancy to various challenges including infection of the uterine mucosa, while accommodating the invasion of semiallogenic trophoblast.

## Summary and Future Questions

Uterine ILCs have proved challenging to study because, although they share characteristics with ILCs in other tissues, they have distinctive phenotypes and functions. The factors responsible for this in the non-pregnant endometrium and the decidual microenvironment remain poorly understood. Through the application of scRNAseq combined with mass and flow cytometry we now have a better understanding of which ILCs are present before and during pregnancy (**Figures 4C**, **5** and **Table 3**). In first trimester decidua the predominant ILCs are dNK1, approximately two fold higher than either dNK2 or dNK3/ieILC1s, and the overall composition most resembles mucosal tumours. Our current understanding of uterine ILCs and their responsiveness is summarised in **Figure 9**. Many experiments investigating uNK functions were carried out prior to the discovery of other uILCs, which would have contaminated these preparations. Some of these questions will need to be revisited to establish the functions of different uILC subsets at each stage of the reproductive cycle. A key new finding has been the identification of three distinct decidual NK subsets, dNK1-3. Much of the existing dNK literature focuses on KIR-expressing dNK which are largely dNK1; the properties and functions of the dNK2 and dNK3 subsets remain to be determined, with dNK3 resembling ieILCs in other tissues. These findings raise a number of questions for future basic and clinically applied research regarding uILCs and uNK cells in particular:

1. **What NK and ILC subsets are present and how can they be identified?**
  Identification of clusters in high dimensional datasets often relies on ‘emergent’ properties where subtle differences across many markers in combination distinguish a subset (143). For human uILCs there is no consensus on simple 2D gating strategies to identify subsets across all the stages of the reproductive cycle. Further work is required to reliably identify and analyse the phenotype and functions of uILC subsets in endometrium and throughout pregnancy.
2. **Where are the NK and ILC subsets located and how does this change?**
  Recent mouse data suggests that the composition of mouse uILCs changes throughout reproductive life and that some uILCs are concentrated at discrete locations. Understanding where human and mouse uILC and uNK subsets localise in the endometrium and during pregnancy will be essential to understanding their functions.
3. **What are the origins of NK and ILCs in the uterus?**
  ILC development in different tissues appears to have divergent pathways as demonstrated by the effects of Nfil3 deficiency on trNK and cNK subsets in the mouse uterus. Which subsets develop *in situ* and which arise from circulating precursors in the human uterus can be addressed by analysing uILCs from patients with bone marrow or uterine transplants with HLA-mismatched donors (74, 144). ILCs also demonstrate considerable plasticity and the relationships between uNK subsets and other uILCs is unclear, although recent evidence suggests that dNK2 can convert to cells resembling dNK1 *in vitro* (76).
4. **What roles do NK and ILC play in repair and homeostasis of the uterine mucosa?**
  While dNK1 appear to be specialised for interactions with EVT through KIR, the roles played by other uNK and uILC subsets is poorly understood in physiological or pathological situations. It will be important to understand how they contribute to mucosal integrity, metabolism, tissue remodelling and antipathogen responses in endometrium and decidua.
5. **Is the huge diversity of phenotypes within known subsets biologically meaningful?**
  In addition to subset discovery, mass cytometry has revealed a previously unappreciated diversity even within well characterised subsets. Lin−CD56+ uNKs exhibit comparable diversity to that reported in pbNK with thousands of related phenotypes defined by stochastic NK receptor expression (**Figure 6**). Determining if this population level variation is biologically meaningful and whether it affects functions such as embryonic implantation and pregnancy outcome is a future challenge.
6. **Do uNK have memory?**
  uILC subsets with a specific phenotype may expand in response to pregnancy or pathogens as previously observed in pbNK (44, 57, 81), where some NKG2C+ pbNK expand in response to CMV infection. How these adaptive NK responses influence pregnancy outcome or pathology will be an important future research question.
  In summary, current evidence suggests that uNK and uILCs are important in supporting aspects of normal placental development in both humans and mice, but the effects of NK or ILC deficiency are nuanced. Loss of functional uNK or uILCs is associated with increased incidence of foetal growth restriction, pre-eclampsia or premature labour in murine models. In humans, these can have life-long consequences including increased cardiovascular and neurological disease (145). Genetic studies of large human cohorts support the idea that variants known to alter NK responsiveness are associated with increased risks of pregnancy disorders (110–112, 116). Understanding how uNK and other uILCs contribute to disorders of implantation and pregnancy will be a major challenge for future studies. Knowledge of subset functions will allow accurate assessment of changes in clinical pathology and infertility and may pave the way for targeted immunotherapies.

**Table 3:** Summary of NK and ILC populations in human first trimester decidua.

Cell Type	Subset	Phenotype	Frequency^1^	Comment	Key References
NK and ILC1s	dNK1	CD49a+ KIR^hi^ NKG2A^hi^ LILRB1^hi^ CD39^hi^	~55%	Receptor profile suggests direct interactions with EVT	(39, 40, 74)
	PTdNK	NKG2C^hi^ LILRB1^hi^	Not detected	CD56+CD3-NKG2C^hi^ primiparous ~3% multiparous ~17% May be expansions of specific dNK1 subsets?	(57)
	dNK2	CD49a+ KIR^lo^ NKG2A^hi^ CD103^lo^	~15%	Distinct receptor profile from dNK1, but do have receptors for ligands on EVT	(39, 40)
	dNK3/ieILC1	CD49a+ CD69^hi^ CD103^hi^ NKG2D^hi^ CD161^hi^ KIR^lo^ NKG2A+/- NKp44+/−	~15%	dNK3 resemble ieILC1s as identified in many tissues.	(33, 36, 37, 39, 40, 62, 63)
	dNKp	CD69^lo^ Ki-67^hi^ NKG2A^hi^ KIR+/−	~5%	KIR profile suggests these are mainly proliferating dNK1	(39, 40, 50)
	pbNK	CD49a^lo^ CD16+/− CD57+/−	<5%	Contaminants from blood. CD56+ CD16+ cells are rare in decidua by immunohistochemistry	(17, 39, 40)
	Classical ILC1s	CD56− CD117− CD127+	Not detected	Identified at very low frequencies?	(36)
ILC2	ILC2	Disputed	Not detected	Expression of CRTH2 not detected in decidua. Lin-CD56-GATA3+ reported in term decidua	(35, 36, 39, 40, 64)
ILC3	NCR+	CD127^hi^ CD117^hi^ CD94− NKp44^hi^	~2%		(34–37, 39, 40)
	NCR-/LTi-like	CD127^hi^ CD117^hi^ CD94− NKp44^lo^	~2%	NCR-ILC3s are phenotypically very similar to LTis	

Frequencies (%) as detected by Huhn et al. (39), using CD45+HLADR−CD14−CD19−CD3− (Lin−) cells as the parent population. Samples are fresh (not cryopreserved).

## Author Contributions

Conceptualisation: OH, FC, LE, AM, and AS. OH and XZ undertook additional analysis and interpretation of published data. Writing: AS, OH, AM, and FC drafted the manuscript and LE and XZ revised and amended it. Visualisation: AS, OH and XZ developed original figures. All authors contributed to the article and approved the submitted version.

## Funding

AS and LE are funded by the MRC (grant number: MR/P001092/1). This work was also supported by the Wellcome Trust (Grant 200841/Z/16/Z to FC and AM), the Centre For Trophoblast Research (XZ) and the Cambridge NIHR BRC Cell Phenotyping Hub (FC). OH was supported by an AstraZeneca-MedImmune-Cambridge PhD fellowship. The funder was not involved in the study design, collection, analysis, interpretation of data, the writing of this article or the decision to submit it for publication.

## Conflict of Interest

The authors declare that the research was conducted in the absence of any commercial or financial relationships that could be construed as a potential conflict of interest.

## Abbreviations

Uterine NK (uNK) and uILC collectively refer to either NK or ILC cells respectively in both non-pregnant (endometrium) or pregnant (decidua) uterine tissue.
